# Pharmacokinetic-pharmacodynamic target attainment analyses as support for meropenem dosing regimens in critically ill adult and elderly patients with *Pseudomonas aeruginosa* infections

**DOI:** 10.3389/fphar.2025.1643553

**Published:** 2025-09-26

**Authors:** Ruyu Tao, Sumyeut Chan, Jinxingyi Wang, Xianhe Wang, Lei Shi, Xue Lan, Ling Chen, Xiaohui Mu

**Affiliations:** ^1^ Center of Laboratory Medicine, The Second Affiliated Hospital of Guizhou Medical University, Kaili, Guizhou, China; ^2^ School of Pharmaceutical Sciences, Sun Yat-Sen University, Guangzhou, China; ^3^ Department of Pharmacy, The Second Affiliated Hospital of Guizhou Medical University, Kaili, Guizhou, China; ^4^ Department of Pathology, The Second Affiliated Hospital of Guizhou Medical University, Kaili, Guizhou, China; ^5^ Department of Reproductive Medicine, Anshun People’s Hospital, Anshun, Guizhou, China

**Keywords:** pharmacokinetics, meropenem, target attainment analyses, critically ill patients, *Pseudomonas aeruginosa*

## Abstract

**Background:**

Critically ill patients often undergo profound pathophysiological changes that alter the pharmacokinetics (PK) of hydrophilic antibiotics such as meropenem. These changes, combined with bacterial susceptibility, may reduce antimicrobial efficacy. This study aimed to optimize meropenem dosing regimens for adult and elderly critically ill patients with Pseudomonas aeruginosa infections using a model-informed approach.

**Methods:**

Limited PK sampling and therapeutic drug monitoring (TDM) data were analyzed to develop a population PK model. Covariates were evaluated, and Monte Carlo simulations (n = 1,000 virtual patients) were conducted to assess alternative dosing strategies, including 0.75–3 g/day (0.5 h infusion) and 0.5–1.5 g every 6–8 h. Probabilities of target attainment (PTA; 40% fT > MIC ×4 and 100% fT > MIC ×4) and cumulative fraction of response (CFR) were calculated.

**Results:**

A one-compartment model with age-adjusted distribution volume best described the data. Population PK estimates were 4.68 L/h for clearance and 4.47 L for distribution volume, both with <50% variability. Age was a significant covariate, with Vd increasing progressively with advancing age. Simulations showed that 750 mg loading followed by 500 mg every 6 h achieved ≥90% CFR in 90-year-old patients, whereas 1 g loading plus 3 g/day (four times daily) was optimal for 40–60-year-old adults.

**Conclusions:**

Age-related increases in meropenem distribution volume necessitate tailored dosing regimens in critically ill patients. More frequent administration (q6h) improved CFR, particularly in elderly populations. Model-informed precision dosing may enhance treatment success against P. aeruginosa infections in intensive care settings.

## 1 Introduction

The emergence of *Pseudomonas aeruginosa* infections in critically ill patients remains a pressing clinical issue, with persistently high mortality and morbidity rates. This problem is exacerbated by the growing threat of antimicrobial resistance, which is often driven by suboptimal drug concentrations, highlighting the critical need for effective antibiotic exposure (Tam et al., 2005). Ensuring proper antibiotic dosing is of utmost importance, particularly for high-priority pathogens such as *P. aeruginosa* (Tacconelli et al., 2018). Prolonged infusion durations represent one of the key strategies for optimizing antibiotic therapy, particularly in the context of beta-lactam antibiotics. Research has shown that extending the infusion time enhances the achievement of pharmacokinetic/pharmacodynamic (PK/PD) targets. This strategy has increasingly become a standard approach in managing severe infections, reflecting its efficacy in improving therapeutic outcomes (Cilloniz et al., 2021; Anh Quan et al., 2022; Craig, 1998).

Meropenem, a carbapenem antibiotic, belongs to the beta-lactam class and is known for its structural resilience against many beta-lactamases due to its unique carbapenem ring. It exhibits a time-dependent bacterial killing mechanism, meaning that optimal activity is achieved when the free drug concentration exceeds the MIC for at least 40% of the dosing interval (40% *T > MIC*) (Craig, 1998). Generally, a f_T.MIC_ of at least 40%–50% of the dosing interval is considered sufficient, though critically ill patients may require a higher target to ensure adequate therapeutic efficacy (Ariano et al., 2005; McKinnon et al., 2008). However, achieving the appropriate therapeutic target in this population is a challenging task for clinicians, particularly in intensive care unit (ICU) settings, due to pathophysiological changes related to sepsis that can significantly alter the pharmacokinetics of meropenem. These changes, including variations in the volume of distribution (Vd) and total drug clearance, result in considerable fluctuations in plasma concentrations, complicating dosage optimization.

Meropenem is frequently administrated in critically ill patients suffering from severe infections, underscoring the need to understand its pharmacokinetics in this specific population to devise an effective dosing regimen. Research indicates that the meropenem pharmacokinetics in critically ill patients differ significantly from those in healthy individuals (Binder et al., 2013). In the context of critical illness, hemodynamic instability can profoundly influence meropenem’s pharmacokinetics. Factors such as increased Vd (e.g., in cases of fluid retention) or altered renal elimination (e.g., in renal insufficiency or augmented renal clearance) can increase non-renal drug clearance, which in turn affects drug exposure and therapeutic outcomes (Boucher et al., 2006).Failure to achieve the optimal PK/PD targets significantly increases the risk of treatment failure and promotes the development of drug-resistant organisms (Binder et al., 2013).

Although the pharmacokinetics of meropenem in critically ill patients have been extensively analyzed, most existing research has been performed on relatively small sample cohorts (Roberts et al., 2009; Crandon et al., 2011; Mathew et al., 2016; Tsai et al., 2016), which may limit the robustness and applicability of the results. Additionally, there is still a lack of comprehensive clinical data on the correlation between meropenem exposure and therapeutic outcomes. In light of these gaps, the purposes of the present study were threefold: to define the population pharmacokinetic estimates of meropenem in critically ill patients suffering from *P. aeruginosa* infections, to investigate the association between meropenem exposure and therapy outcomes, and to assess various dosing regimens that effectively achieve the PK/PD targets.

## 2 Materials and methods

### 2.1 Ethical approval and patient eligibility

Ethical approval for this study was obtained from the Hospital Ethics Committee of the Second Affiliated Hospital of Guizhou Medical University (No: 2023-LS-221). Patients were included if they were admitted to the surgical ICU and were receiving meropenem as a continuous infusion. Exclusion criteria included individuals under 18 years of age, as well as those undergoing renal replacement therapy (RRT) or extracorporeal membrane oxygenation (ECMO) during the course of meropenem administration. Meropenem dosing regimens were adjusted according to the measured creatinine clearance (CrCL). A loading dose of 2 g was administered over 30 min, immediately followed by continuous infusion of meropenem. Blood samples were collected from patients admitted to the surgical ICU between 1 March 2023, and 1 October 2024, after a minimum of 6 h of meropenem therapy. In addition to blood samples, data on biochemistry results (albumin, transaminase levels, renal function, etc.), demographic characteristics (age, weight, sex, etc.), SOFA scores, and APACHE II scores at admission were prospectively recorded at baseline and at the time of blood sampling. Detailed information regarding dosing (including date, time, dosage, and duration) and the timing of blood collection were also documented. Specially, for a 24-h continuous infusion, meropenem was therefore reconstituted and prepared three times per day.

### 2.2 LC-MS/MS analysis for meropenem

The analysis of meropenem was conducted using liquid chromatography-mass spectrometry (LC-MS/MS). Reagents such as water, methanol (LC-MS grade), acetic acid, and ammonium acetate were purchased from Tedia Company Inc. (Beijing, China) and Sigma-Aldrich (Steinheim, Germany), while meropenem-D_6_, an internal standard, was acquired from Toronto Research Chemicals (Ontario, CA). The mobile phase used in high-performance liquid chromatography (HPLC) comprised two components: phase A consisted of water containing 2 mM ammonium acetate and 0.1% acetic acid, while phase B consisted of methanol. Sample preparation for plasma and dialysate was performed according to the method described by Koal et al. (2006). To each sample, 10 µL of methanolic meropenem-D6 (target concentration: 1 mg/mL) was added as an internal standard.

Chromatographic separation was achieved using an Acquity BEH C18 column (2.1 mm × 50 mm, 1.7 μm particle size, Waters, United States), coupled with a guard cartridge. The separation gradient was implemented as follows: initially, the mobile phase consisted of 95% phase A and 5% phase B for 0 min. This was gradually changed to 10% phase A and 90% phase B at 4 min. Subsequently, a second gradient was applied, transitioning from 10% phase A and 90% phase B at 4 min to 95% phase A and 5% phase B at 6 min. The system was then equilibrated for 3 min before the next injection. Mass spectrometric analysis was performed using a TSQ Quantum Ultra triple-quadrupole mass spectrometer (Thermo Fisher Scientific Inc., Boston, United States) operating in positive electrospray ionization (ESI) mode. The parameters included a spray voltage of 4.0 kV, capillary temperature of 350 °C, vaporizer temperature of 280 °C, sheath gas pressure of 20 psi, auxiliary gas pressure of 5 psi, collision gas pressure of 2.0 mTorr. The transitions of the precursors to the product ions were m/z 384.1 → m/z 141.2 and m/z 254.2 for meropenem, and m/z 390.1 → m/z 147.2 for meropenem-D_6_. The limit of quantification (LOQ) was 0.1 mg/L for plasma, with the assay demonstrating linearity from 0.1 to 100 mg/L. The validation of this bioassay was evaluated, and meet requirements as per guidelines for analysis of biological samples.

### 2.3 Pharmacokinetic modeling

PK modeling was performed using Phoenix NLMN™ software (version 7.0, Certara L.P Pharsight, St. Louis, MO, United States), employing a systematic three-step approach: (i) selection of the basic model, (ii) identification of relevant covariates, and (iii) validation of the model. The first-order conditional estimation method with interaction was applied for parameter estimation. Interindividual variability (IIV) was modeled using a log-normal distribution, while residual variance was assessed through additive, proportional, and combined error models. The performance of the model was evaluated using several criteria: (i) the reduction in the objective function value (−2 log likelihood), (ii) graphical comparison of predicted *versus* observed concentrations, and (iii) examination of changes in the standard error of parameter estimates. These assessments ensured the reliability and robustness of the final model (Li et al., 2022).

In the second step, potential covariates, including both demographic and clinical variables, were evaluated for their inclusion in the basic model. The influence of these covariates on model parameters was assessed using graphical methods and generalized additive models. Continuous covariates were examined as either proportional or power functions, whereas categorical variables were incorporated using an appropriate mathematical equation P_j_ = PPOP_COV · (1–Covi), where P_j_ is the PK parameter for patient j, Covi is the covariate, and PPOP is the typical value of the PK parameter. Covariates that improved model fit were retained, based on biological plausibility, graphical concordance, and statistical tests.

### 2.4 Model evaluation

To assess the stability and performance of the final model, a nonparametric bootstrap resampling technique was employed (Mathew et al., 2016). The original dataset was resampled at the subject level to produce 1,000 new replications. These resampled datasets were then used to calculate the 2.5th and 97.5th percentiles of the model parameters. If the model was valid, the parameter estimates from the original dataset should closely align with the median and fall within the 2.5th and 97.5th percentiles of the distributions generated through resampling (Li et al., 2022).

The performance of the final model was further assessed using a prediction-corrected visual predictive check (pc-VPC), a technique that integrates virtual predictions and observed data derived from Monte Carlo simulations. In this approach, the percentiles of the simulated data were compared with the corresponding percentiles of the observed data, thereby providing a thorough evaluation of the model’s predictive accuracy.

### 2.5 Dosing simulations

Monte Carlo simulations were conducted to simulate concentration-time profiles for 1,000 patients per dosing regimen, using the final population PK parameters. Different loading dosages (LD) with four bolus maintenance dosing (MD) regimens were simulated as follows: a. 2000 mg LD+ 1,000 mg every 8 h; 2000 mg LD+ 1,500 mg every 8 h; 2,500 mg LD+ 2000 mg every 8 h; 3,000 mg LD+ 2,500 mg every 8 h b. 750 mg LD+ 500 mg every 6 h; 1,000 mg LD+ 750 mg every 6 h; 1,500 mg LD+ 1,000 mg every 6 h; 1,500 mg LD+ 1,500 mg every 6 h.

The simulations incorporated patient age groups of 20, 40, 60, and 90 years. For each regimen, the proportion of patients achieving the PK/PD target of 40% fT > MIC × 4 was calculated. MIC distributions ranging from 0.008 to ≥64 mg/L were considered as the pharmacodynamic (PD) parameter for meropenem, following the Clinical and Laboratory Standards Institute (CLSI) Executive Standards for Antimicrobial Susceptibility Testing (Humphries et al., 2021). To ensure the convenience of clinical application, MIC valuesof 0.25, 0.5, 1, 2, 4, 8, and 16 mg/L were selected based on a study published previously (Hou et al., 2024). PK/PD target values were set as 40%fT > MIC × 4 and 100%fT > MIC × 4 (Zhang et al., 2024; Roberts et al., 2025), to evaluate both the therapeutic efficacy and the potential for resistance suppression of meropenem. These target values were selected on the basis of previously published criteria for carbapenem efficacy and resistance prevention (Zhang et al., 2024). A clinical susceptibility breakpoint was set to 2 mg/L, consistent with meropenem breakpoints reported by McKinnon et al. (2008). For each dosing regimen, the PTA was determined and subsequently utilized to calculate the CFR against the bacterial population, following approaches described in earlier studies (Koomanachai et al., 2016).

### 2.6 Statistical analysis

Statistical analysis was performed using SPSS software (version 20 for Macintosh, IBM SPSS Statistics, United States). Categorical variables were expressed as both absolute and relative frequencies, whereas continuous variables were reported as medians with their respective ranges. For comparisons involving normally distributed variables, a two-tailed Student’s t-test was applied, while the Mann-Whitney U test was used for non-normally distributed data. The chi-square test or Fisher’s exact test, depending on the context, was employed for categorical variables. A significance level of p ≤ 0.05 was considered statistically significant for all analyses.

## 3 Results

### 3.1 Study population

Data from 144 adults and elderly patients (mean (SD) age 60.63 (18.37) years, 58 males and 86 females) in Intensive Care Unit were initially recruited between 1 March 2023 and 1 October 2024, for this study. A summary of the demographic characteristics of the participants is provided in Table 1. The median (IQR) value of trough concentration was 10.40 (3.39,22.53) mg/L. Patients received 2000 mg of meropenem dosing as an initial the loading dose, followed by daily maintenance dose of 500 or 1,000 mg (q8h/q12h).

**TABLE 1:** Demographic data and characteristics of the enrolled patients.

Characteristics	Critically ill patients (n = 144)
Gender	
Males, n (%)	86 (59.72%)
Females, n (%)	58 (40.28%)
Age (years)	63.50 (46.50,76.0)
Serum albumin concentration (mg/L)	32.00 (17.90,39.80)
Serum creatinine concentration (μmol/L)	40.00 (22.00,62.75)
Loading meropenem dose [g/day, n (%)]	2.0 (100.00%)
Maintenance meropenem dose (g)	0.5 (100.00%)
0.5g, n (%)	45 (31.25%)
1.0g, n (%)	99 (68.75%)
Dosing intervals	
q8h, n (%)	85 (59.03%)
q12h, n (%)	59 (40.97%)
Meropenem concentration (mg/L)	10.40 (3.39,22.53)

### 3.2 Pharmacokinetic model building

A total of 144 blood samples were obtained from the enrolled patients. Among these, 5 samples (3.47%) had concentrations below the LOQ. For those observations that were considered normally distributed, values below the LOQ were substituted with random values drawn between negative infinity and the lower LOQ, referred to M3 method, as described previously (Palaparthy et al., 2019; Li et al., 2022). Consequently, all detectable concentrations, including those below the LOQ, were treated as continuous data for population pharmacokinetic (popPK) analysis.

The pharmacokinetic profiles of meropenem was modeled using a one-compartment model with first-order elimination. This model demonstrated a significantly lower objective function value (OFV) of 873.81, compared to the two-compartment model, which yielded an OFV of 928.18. The model described the pharmacokinetics of meropenem in critically ill patients, with the corresponding parameter estimates provided in Table 1. Residual variability was adequately accounted for using an additive error model, as the combined proportional and additive error model did not offer a significant improvement. Furthermore, IIV in CL and Vd decreased from 8.91% to 4.22% in the base model to 4.50% and 1.60% in the final model, respectively. Residual variability showed a minor increase from 0.72 in the base model to 0.74 in the final model. These findings highlight the refinement and improved fit of the model with respect to both individual and residual variabilities.

### 3.3 Covariate analysis

**FIGURE 1:**
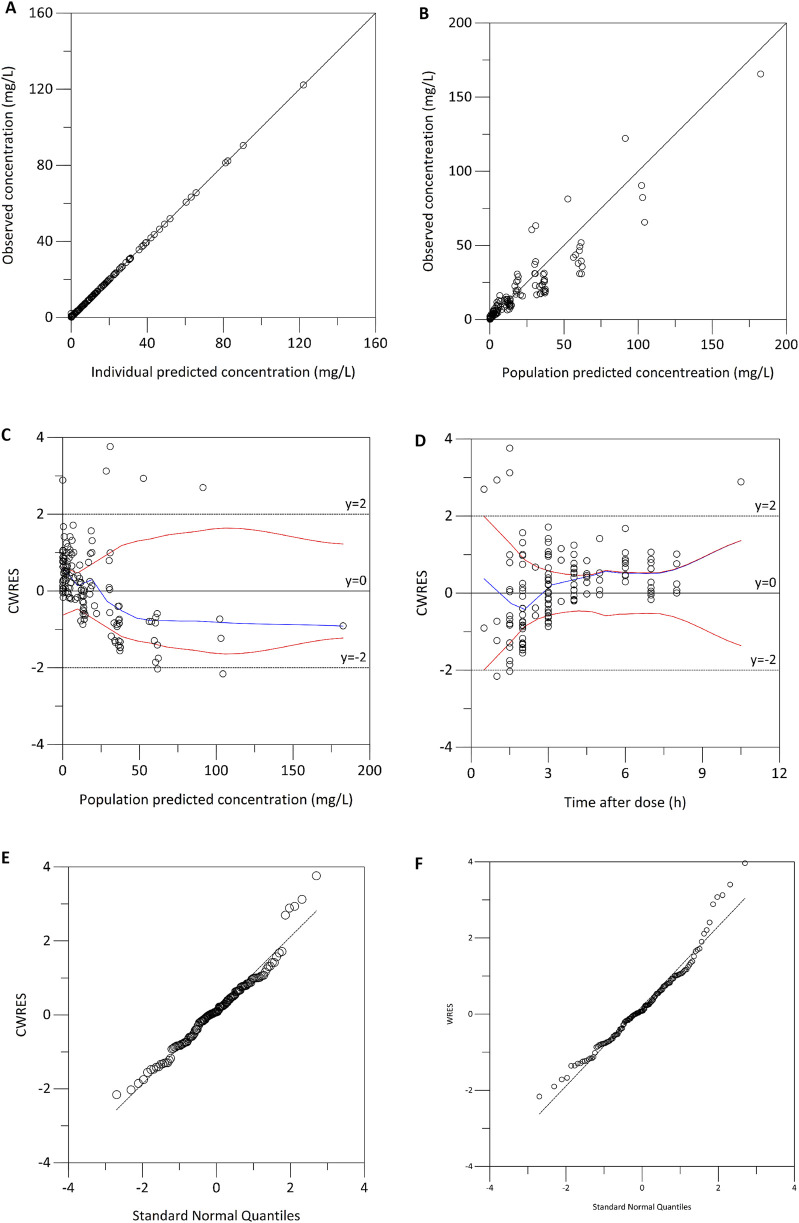
Model diagnostics for the final meropenem pharmacokinetic model. **(A)** Observed concentrations *versus* individual predicted concentrations. **(B)** Observed concentration *versus* population predicted concentration. **(C)** Conditional weighted residuals (CWRES) *versus* population predicted concentrations. **(D)** CWRES *versus* time after dose. **(E)** Normal quantile-quantile plot of CWRES. **(F)** Normal quantile-quantile plot of weighted residuals.

During the covariate analysis, potential clinical and demographic factors, including age, body weight, sex, serum creatinine, and renal function, were systematically evaluated for their impact on meropenem clearance and volume of distribution. In the forward selection phase, age was initially included in the base model for both Cl and Vd, leading to a significant reduction of 10.36 points in the objective function value (OFV). The inclusion of estimated glucose (GLU) also resulted in a modest, but statistically significant, decrease of 2.16 points in the OFV. However, during the backward elimination step, estimated glomerular filtration rate (eGFR) was excluded from the model. And ultimately, age was the only covariate retained which had the most substantial impact on the meropenem Vd, contributing the largest decrease in OFV among all potential covariates. Compared with the base model, the final PK model led to a significant decrease of Akaike Information Criterion (−1.02%) and Bayesian Information Criterion (−1.33%). Estimates of the shrinkage for random variabilities of Vd and CL were 0.35 and 0.12, respectively. The condition number was 10.93. Model diagnostics, as illustrated in Figure 1, indicated that the final model for meropenem pharmacokinetics demonstrated an acceptable goodness-of-fit. In this final model, the individual pharmacokinetic parameters were optimally described using the following equations:

$$CL\left(L/h\right)=4.68\times e^{0.045}$$

$$Vd\left(L\right)=4.47\times {\left(\frac{Age}{63.5}\right)}^{0.29}\times e^{0.016}$$

**FIGURE 2:**
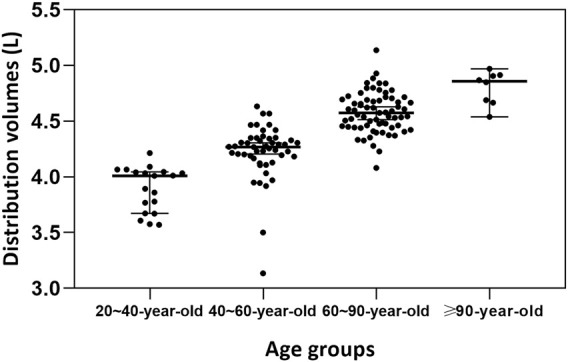
Age group comparison of the estimated age-normalized volume of distribution (Vd) at steady state.

The median (range) values of the estimated age-normalized Vd at steady state were 4.41 (4.21–4.62) L. As illustrated in Figure 2, the age-normalized Vd increased with advancing age, indicating a clear trend across different age groups.

### 3.4 Model validation

**FIGURE 3:**
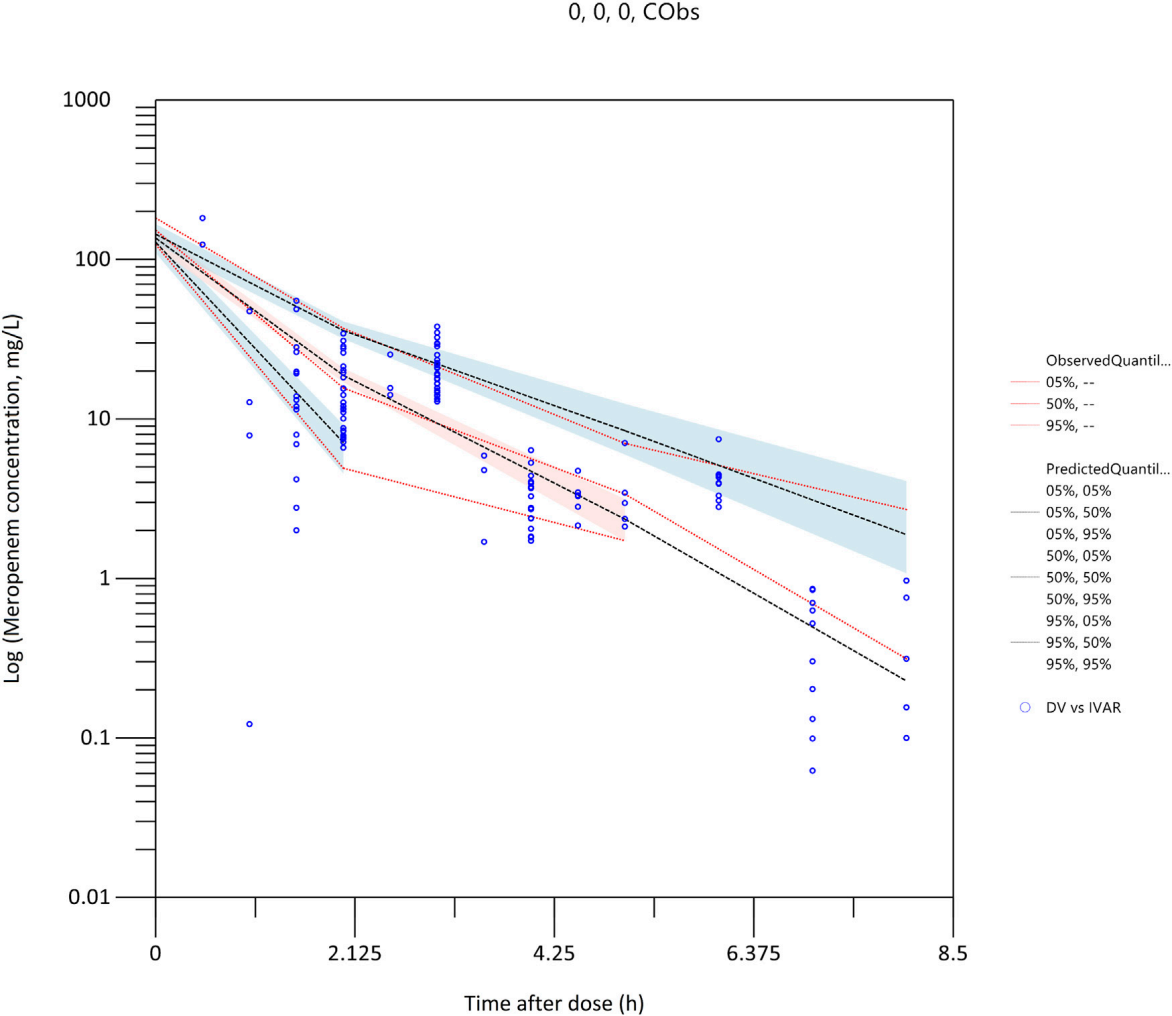
Prediction-corrected visual predictive check (pcVPC) plot for the population pharmacokinetic model of meropenem.

The robustness and stability of the final pharmacokinetic model were evaluated using both bootstrapping (Table 2) and prediction-corrected visual predictive checks (Figure 3). In the bootstrapping analysis, the median of the fixed-effects parameter estimates obtained from the resampled datasets was within 5% of the population parameter estimates derived from the original dataset for all parameters, suggesting high reliability and accuracy. Moreover, the comparison between predicted and observed meropenem concentrations demonstrated excellent concordance. Specifically, the 5th, 50th, and 95th percentiles of the predicted concentrations were closely aligned with those observed in the clinical data. This alignment further supports the model’s predictive accuracy. Model diagnostics confirmed that the final meropenem pharmacokinetic model exhibited a satisfactory goodness of fit. Figure 1 shows that there were no discernible trends between the population predicted concentrations (PRED) and the conditional weighted residuals (CWRES), nor was there any correlation between time and CWRES. Additionally, as presented in Table 2, the median values of the parameter estimates obtained through bootstrapping were consistent with those from the final model. This consistency further indicates the stability of the model and reinforces the reliability of the parameter estimates derived from the population pharmacokinetic analysis. Finally, the results from the pcVPC, shown in Figure 3, revealed that the 5th, 50th, and 95th percentiles of the observed concentrations fell within the 95% confidence interval of the predicted concentrations, thereby demonstrating the model’s predictive accuracy and overall adaptability to the data.

**TABLE 2:** Population pharmacokinetic parameters of meropenem and bootstrap validation.

Parameters	Final model (n = 144)		Bootstrap (n = 1,000)	
	Estimate	*RSE* (%)	Median	95% *CI*
CL (L/h)	4.68	8.38	4.71	(3.36, 6.11)
Vd (L)	4.47	13.23	4.55	(3.89, 5.64)
θ_Age,V_	0.19	35.62	0.18	(-0.04, 0.35)
Between-subject variation				
ω^2^_CL_	0.045	15.90	0.044	(0.030, 0.058)
ω^2^_Vd_	0.016	43.66	0.012	(-0.009, 0.034)
Within-subject variation				
σ_additive_ (mg/L)	0.74	24.20	0.72	(0.01, 1.07)

### 3.5 Probability of target attainment of different dosing regimens of meropenem on each minimum inhibitory concentration of *Pseudomonas aeruginosa*

**FIGURE 4:**
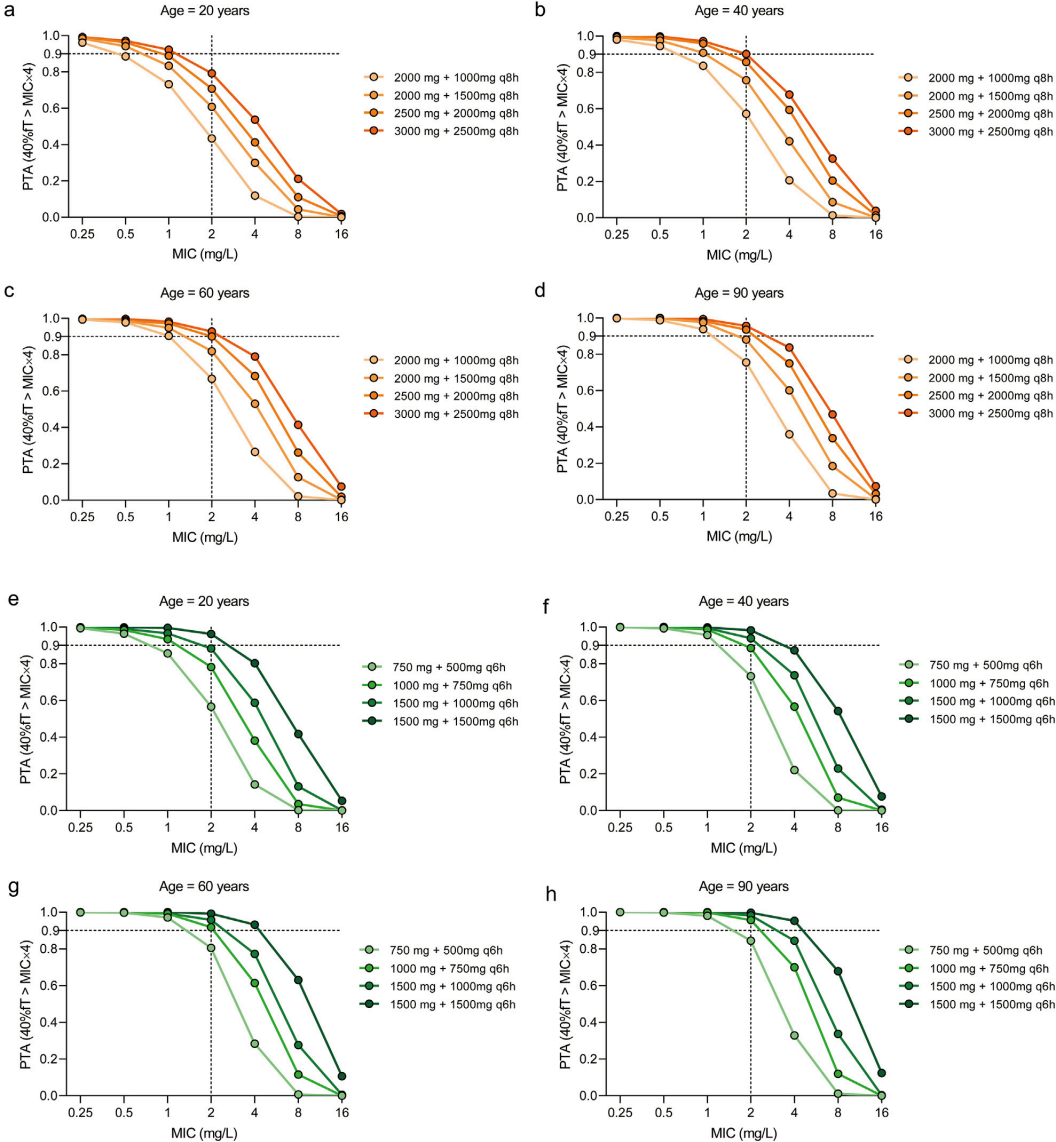
Probability of target attainment (PTA) of meropenem dosing regimens across different age groups and MIC values. The target pharmacokinetic/pharmacodynamic values was set as 40%fT > MIC×4. The PTAs for patients aged 20 years **(a,e)**, 40 years **(b,f)**, 60 years **(c,g)** and 90 years **(d,h)** with four dosing regimens. The dosing intervals for the regimens were 8 h **(a–d)** and 6 h **(e–h)**, respectively.

Monte Carlo simulations (Figure 4) demonstrated that, in critically ill patients infected with *P. aeruginosa*, all meropenem dosing regimens achieved a PTA exceeding 90% when the MIC was 0.25 mg/L in both every-8-h (Figures 4a–d) and every-6-h (Figures 4e–h) dosing regimen. At an MIC of 0.5 mg/L, all meropenem dosing regimens achieved a PTA >90% at each age group, while escalation of the meropenem MD dose to 1,500 mg every 8 h was required to maintain adequate exposure in the 20-year-old group (Figure 4a). For an MIC of 1 mg/L, a regimen of LD 3000 mg and MD 2500 mg every 8 h achieved a PTA of 92.20%, 97.20%, 98.20% and 99.40% for the 20-year-old group, the 40-year-old group, the 60-year-old group and the 90-year-old group, respectively. When the MIC increased to 2 mg/L, shortening the dosing interval to 6 h was necessary, yielding PTAs exceeding 90% in each age group. Using an MIC of 2 mg/L as the clinical susceptibility breakpoint, the regimen of 1,500 mg LD followed by 1,000 mg q6h was the most appropriate option for patients aged 20 years, and 1,000 mg LD followed by 750 mg q6h for 40–90 years, as it consistently achieved PTAs above 90%. At MIC values of 4 mg/L, only the regimen of 1,500 mg LD followed by 1,500 mg q6h attained a PTA greater than 90% in patients aged ≥60 years old. At MIC values ≥ 8 mg/L, however, none of the simulated regimens attained a PTA greater than 90%.

**FIGURE 5:**
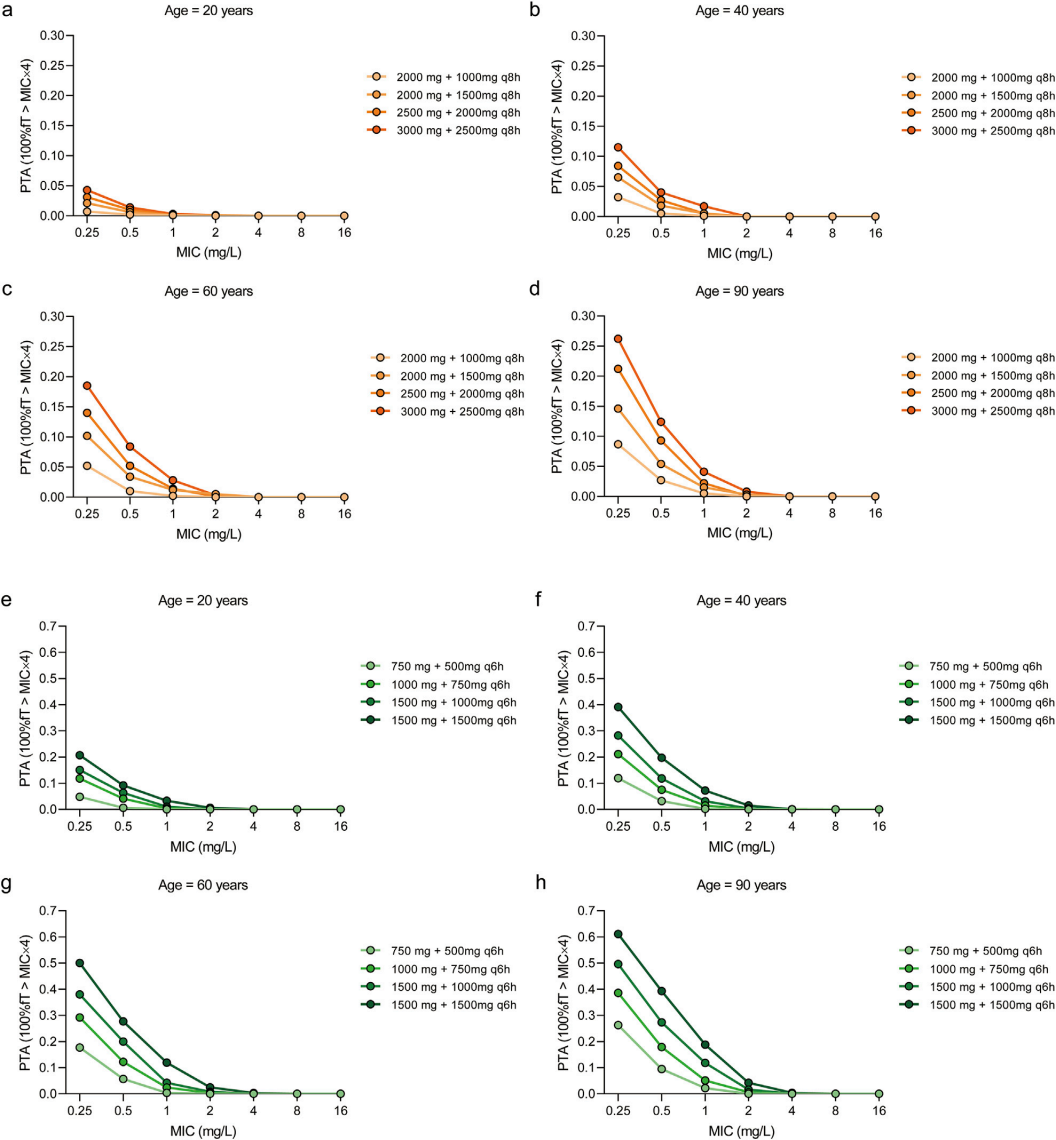
Probability of target attainment (PTA) of meropenem dosing regimens across different age groups and MIC values. The target pharmacokinetic/pharmacodynamic values was set as 100%fT > MIC×4. The PTAs for patients aged 20 years **(a,e)**, 40 years **(b,f)**, 60 years **(c,g)** and 90 years **(d,h)** with four dosing regimens. The dosing intervals for the regimens were 8 h **(a–d)** and 6 h **(e–h)**, respectively.

When the more stringent pharmacodynamic threshold of 100% fT > MIC × 4 was applied to assess resistance suppression (Figure 5), simulations indicated that administering meropenem at 1,500 mg every 6 h in patients aged over 60 years achieved a PTA of 50.00% at an MIC of 0.25 mg/L. For MICs ≥0.5 mg/L, no regimen achieved PTA values above 50%; among the tested regimens, LD 1500 mg and MD 1500 mg every 6 h provided the highest relative PTA, though it remained below the desired threshold.

### 3.6 Cumulative fraction of response values of different dosing regimens of meropenem on each minimum inhibitory concentration of *Pseudomonas aeruginosa*

When the PK/PD target was defined as 40% fT > MIC×4, the CFR for the regimens consisting of LD of 1,500 mg followed by a MD of either 1,000 mg q6h or 1,500 mg q6h exceeded 90% across the population distribution of MICs (Hou et al., 2024) suggesting that both dosing strategies should be prioritized as empirical treatment options for *P. aeruginosa* in 20-year-old patients (Table 3). The regimen of LD 2500 mg + MD 2000 mg q8h, LD 3000 mg + MD 2500 mg q8h, LD 1000 mg + MD 750 mg q6h, LD 1500 mg + MD 1000 mg q6h, and LD 1500 mg + MD 1500 mg q6h achieved CFRs above 90% in 40-year-old and 60-year-old patients. With the exception of the regimen of LD 2000 mg + MD 2000 mg q8h, all other dosing strategies achieved CFR values exceeding 90% in 90-year-old patients. When considering a more stringent PK/PD target,100% fT > MIC × 4, only the regimen of LD 1500 mg + MD 1500 mg q6h achieved the highest relative CFRs of 12.59%, 24.31%, 32.06% and 40.90% for 20-year-old, 40-year-old,60-year-old and 90-year-old patients, respectively (Table 3).

**TABLE 3:** Summary of cumulative fraction of response for tested meropenem dosing regimens against *Pseudomonas aeruginosa*.

Age (years)	Dosing regimens (LD + MD)	CFR %	
		40% fT > MIC × 4	100% fT > MIC × 4
20	2000 mg + 1,000 mg q8h	79.37%	0.41%
	2000 mg + 1,500 mg q8h	84.69%	1.18%
	2,500 mg + 2000 mg q8h	87.36%	1.74%
	3,000 mg + 2,500 mg q8h	88.99%	2.46%
40	2000 mg + 1,000 mg q8h	84.20%	1.73%
	2000 mg + 1,500 mg q8h	88.08%	3.68%
	2,500 mg + 2000 mg q8h	90.74%	4.77%
	3,000 mg + 2,500 mg q8h	91.85%	6.81%
60	2000 mg + 1,000 mg q8h	87.39%	2.85%
	2000 mg + 1,500 mg q8h	89.83%	5.97%
	2,500 mg + 2000 mg q8h	91.47%	8.18%
	3,000 mg + 2,500 mg q8h	92.59%	11.23%
90	2000 mg + 1,000 mg q8h	89.03%	4.93%
	2000 mg + 1,500 mg q8h	91.30%	8.54%
	2,500 mg + 2000 mg q8h	92.36%	12.58%
	3,000 mg + 2,500 mg q8h	93.32%	16.01%
20	750 mg + 500 mg q6h	85.44%	2.54%
	1,000 mg + 750 mg q6h	89.03%	6.67%
	1,500 mg + 1,000 mg q6h	90.67%	8.73%
	1,500 mg + 1,500 mg q6h	93.02%	12.59%
40	750 mg + 500 mg q6h	89.11%	6.56%
	1,000 mg + 750 mg q6h	91.37%	12.14%
	1,500 mg + 1,000 mg q6h	92.36%	16.72%
	1,500 mg + 1,500 mg q6h	93.67%	24.31%
60	750 mg + 500 mg q6h	89.91%	9.90%
	1,000 mg + 750 mg q6h	91.79%	17.11%
	1,500 mg + 1,000 mg q6h	92.56%	23.04%
	1,500 mg + 1,500 mg q6h	94.16%	32.06%
90	750 mg + 500 mg q6h	90.42%	15.23%
	1,000 mg + 750 mg q6h	92.29%	23.30%
	1,500 mg + 1,000 mg q6h	92.97%	31.75%
	1,500 mg + 1,500 mg q6h	94.38%	40.90%

CFR, cumulative fraction of response; LD, load dose; MD, maintenance dose; MIC, minimum inhibitory concentration.

According to previous studies, achieving a target of at least 40%–50% fT > MIC×4 during the dosing interval is generally considered sufficient to ensure adequate therapeutic efficacy in critically ill patients (Ariano et al., 2005; McKinnon et al., 2008). Based on this criterion and the MIC distribution of *P. aeruginosa*, the recommended empirical regimens are LD 1500 mg followed by MD 1000 mg every 6 h for 20-year-old patients, LD 1000 mg followed by MD 750 mg every 6 h for 40–60-year-old patients, and LD 750 mg followed by MD 500 mg every 6 h for 90-year-old patients, thereby safeguarding the antimicrobial efficacy of meropenem across different age groups.

## 4 Discussion

We conducted a population pharmacokinetic study of meropenem in critically ill adult and elderly patients with *P. aeruginosa* infections. To the best of our knowledge, this is the largest cohort study up until now and developed an optimal dosage regimen of meropenem for critically ill patients aged from 18 years and over.

In the current study, a one-compartment PK model with first- order elimination fitted well to depict the PK data of meropenem, consistent with previous studies (Huang et al., 2024; Rancic et al., 2024). However, Boonpeng et al. (2022), Lan et al. (2022), and Usman et al. (2017) found their models to be two-compartment. The typical estimate of CL was 4.68 L/h, was similar with the CL of 4.8L/h (Boonpeng et al., 2022) and 5.71L/h (Usman et al., 2017). Whereas it was lower than the meropenem CL of 9.69 L/h (Huang et al., 2024) and 8.31 L/h (Mathew et al., 2016) in patients with normal renal function. Though the CrCL was not as significant covariate in the final model. This may also be explained by the tendency for increased volume distributions in elderly patients in our study. A notable trend of decreasing CrCL was observed as the age of patients increased, which consequently led to a reduction in the CL of meropenem. In the final pharmacokinetic model, Vd of meropenem was found to be significantly affected by age. While this observation is not widely supported in the existing literature, a plausible explanation may lie in the hydrophilic nature of meropenem (Klotz et al., 1975; Usman et al., 2017). As people grow older, significant changes to the body occur, including an increase in body fat and a reduction in total body water (Usman et al., 2017). Such changes may result in a decreased volume of distribution for hydrophilic drugs, like meropenem, whereas the distribution of lipophilic drugs tends to increase with age (Hanratty et al., 2000; Klotz et al., 1975).

Pharmacokinetic alterations in critically ill patients have been extensively studied and are crucial in determining appropriate drug dosing, particularly for antimicrobial agents. However, a significant challenge remains, as most antimicrobial drugs do not have routine monitoring of drug concentrations or clear parameters for assessing their efficacy and toxicity. As a result, adjusting the dosing of antimicrobial agents in critically ill patients continues to be difficult, primarily due to the need to achieve pharmacodynamic targets amidst the inherent pharmacokinetic variations in this patient population. Studies have shown that critically ill patients may experience altered volume of distribution and clearance, leading to potential suboptimal drug concentrations, which could result in treatment failure or resistance development (Cilloniz et al., 2021). In older ICU patients, there is an increased complexity due to age-related decline in renal function and organ perfusion. Such changes can lead to a prolonged half-life of meropenem, thus requiring dose adjustment to avoid drug accumulation and toxicity (Vardakas et al., 2018). Age-related changes in protein binding also alter drug pharmacodynamics, influencing the free drug concentration available for antimicrobial activity. As a result of these pharmacokinetic differences, personalized dosing strategies must be considered for both young and elderly ICU patients. Herein, since high volume results, in our study, the elderly patients (ages≥ 60-year-old) reached the desired attainment rates of 40% fT > MIC×4 than the adults patients, and increased doses of meropenem, both at loading and maintenance phases, could improve the probability of target attainment.

Based on the simulation results, administration of meropenem using the standard dosing regimen (loading dose 2000 mg + maintenance dose 1,000 mg every 8 h) was insufficient to achieve the desired PK/PD target. To address this, higher doses may be required when the MIC is low (e.g., 0.25–0.50 mg/L as the breakpoint). Additionally, for higher MIC values (e.g., 2 mg/L as the breakpoint for more resistant pathogens, particularly *P. aeruginosa*), shortening the dosing intervals could be an effective strategy. These findings are consistent with recent studies in this field (Usman et al., 2017). An alternative approach to achieve the PK/PD target is to extend the infusion duration (e.g., 3-h infusion) (Kothekar et al., 2020; Venuti et al., 2023; Murinova et al., 2024). However, when using continuous infusion, it is important to note that the stability of meropenem, when mixed with physiological saline or 5% glucose, is limited at room temperature. And it is possible that meropenem could be continuously infused for at least 7 h if temperature does not exceed 22 °C (Fawaz et al., 2019). Therefore, it is essential to adhere to the manufacturer’s guidelines regarding the stability and administration conditions. Based on our analysis, administering the drug four times a day appears to be the most reliable method for ensuring that the PK/PD target is met during treatment. However, this regimen may present logistical challenges due to the frequency of dosing.

Recent publications have addressed meropenem PK/PD optimization in critically ill patients, which emphasized the importance of achieving adequate β-lactam exposure under conditions of altered physiology (Hou et al., 2024). Our work extends these findings in several important ways. By integrating MIC distributions, we estimated CFR with two PK/PD indices, allowing regimen performance to be interpreted within a population-level epidemiologic context rather than solely at clinical breakpoints. Second, through population PK modeling with rigorous covariate analysis, we identified age as the primary determinant of V, enabling age-stratified dosing recommendations for patients aged 20–90 years. Finally, we directly compared intermittent q6h/q8h regimens with continuous infusion while explicitly considering reconstitution frequency to reflect stability constraints in real-world ICU practice.

There are several limitations to our study that should be acknowledged. First, we focused solely on the achievement of the PK/PD target and did not assess the clinical outcomes of treatment. The effectiveness of antibiotic therapy depends not only on the serum concentration of meropenem but also on the concentration at the site of infection, with the MIC of the pathogen playing a critical role in therapeutic success (Themans et al., 2019). Second, the relatively small sample size may restrict the statistical power and precision of parameter estimates. Third, the findings were derived from a single-center cohort, which may limit the external generalizability to other critically ill populations with different demographic or microbiological characteristics. Finally, as with all model-based analyses, certain structural and statistical assumptions were required, and deviations from these assumptions could affect predictive performance. We also did exclude patients undergoing renal replacement therapy, meaning that our dosing recommendations may not be applicable to this specific subgroup.These limitations highlight the need for validation in larger, multicenter cohorts to confirm the robustness and applicability of our conclusions.

## 5 Conclusion

We developed a meropenem population PK model in a cohort of in critically ill adult and elderly patients with *P. aeruginosa* infections. Meropenem V was associated with age and was also increased in elderly patients. We proved the superiority of shorten the dosing intervals in attainment of the PK/PD targets for the treatment of *P. aeruginosa* infections in critically ill patients. Dosing regimens of 750 mg/day administered as loading dose and maintenance doses of 500 mg administered q6h ≥ 90% PTAs for 90-year-old patients. The dosing of 1,000 mg administered as loading dose and 3.0 g maintained via four times a day was found to be accurate for 40–60 -year-old adults.

## Data Availability

The raw data supporting the conclusions of this article will be made available by the authors, without undue reservation.
